# Parkinson’s Disease: Overview of Transcription Factor Regulation, Genetics, and Cellular and Animal Models

**DOI:** 10.3389/fnins.2022.894620

**Published:** 2022-05-04

**Authors:** Ninuo Xia, Deborah E. Cabin, Fang Fang, Renee A. Reijo Pera

**Affiliations:** ^1^Division of Life Sciences and Medicine, The First Affiliated Hospital of USTC, University of Science and Technology of China, Hefei, China; ^2^McLaughlin Research Institute for Biomedical Sciences, Inc., Great Falls, MT, United States

**Keywords:** Parkinson’s disease, transcription factor, α-synuclein, animal models, cellular models

## Abstract

Parkinson’s disease (PD) is one of the most common neurodegenerative disorders, affecting nearly 7–10 million people worldwide. Over the last decade, there has been considerable progress in our understanding of the genetic basis of PD, in the development of stem cell-based and animal models of PD, and in management of some clinical features. However, there remains little ability to change the trajectory of PD and limited knowledge of the underlying etiology of PD. The role of genetics versus environment and the underlying physiology that determines the trajectory of the disease are still debated. Moreover, even though protein aggregates such as Lewy bodies and Lewy neurites may provide diagnostic value, their physiological role remains to be fully elucidated. Finally, limitations to the model systems for probing the genetics, etiology and biology of Parkinson’s disease have historically been a challenge. Here, we review highlights of the genetics of PD, advances in understanding molecular pathways and physiology, especially transcriptional factor (TF) regulators, and the development of model systems to probe etiology and potential therapeutic applications.

## Introduction

The clinical features of Parkinson’s disease (PD), as a shaking or tremor disease, were first described by James Parkinson in 1817 (Goetz, 2011). Subsequent observations and analysis rounded out the clinical picture with the inclusion of rigidity, bradykinesia, postural instability, and non-motor clinical features such as sleep disturbance, cognitive and neuropsychiatric abnormalities and dysfunction of the autonomic nervous system as well as non-neuronal symptoms (Dawson and Dawson, 2003; Bloem et al., 2021). At present, although no therapies have been identified that can slow down or arrest progression of PD, personalized management of the disease that takes into account an array of clinical features has increased quality-of-life following diagnosis (Stoddard-Bennett and Pera, 2019; Bloem et al., 2021).

## Embryonic Development and Loss of Select Neurons

During embryonic development, midbrain dopamine (mDA) neurons arise from the floor plate region of the mesencephalon (Ono et al., 2007). They develop into three anatomically distinct clusters termed the A8, A9, and A10 groups. Neurons in the three groups have axonal projections to different target areas and control different brain functions. The A9 group gives rise to the substantia nigra pars compacta (SNc) and projects to the dorsal striatum. Neurons in the SNc are essential for voluntary movement as well as motor learning, habit formation, goal-directed action selection, and exploration (Chase, 2004; Tricomi et al., 2009; Jin and Costa, 2010; Costa, 2011; Schiemann et al., 2012; Everitt and Robbins, 2013). Irreversible loss of these neurons is one of the main pathological hallmarks of PD, making it the second most common neurodegenerative disease [after Alzheimer’s disease (AD)]. Current treatments for PD, including drug therapies and deep brain stimulation, only relieve the symptoms and will lose potency as the disease progresses. Treatments that replace lost neurons and restore neuron functions are needed to reverse or slow down the progression of the disease, thus there has been a focus on use of stem cell systems such as induced pluripotent stem cells (iPSCs) for potential neuron replacement therapies (Stoddard-Bennett and Pera, 2019; Bloem et al., 2021).

## Genetics of Parkinson’s Disease

Since the identification of the first gene linked to PD in 1997, more than 20 genes and loci have been identified. Together, these monogenic causes for PD are believed to account for approximately 5–25% of cases, an estimate that differs between different studies and between different study populations (Klein and Westenberger, 2012; Bloem et al., 2021). In addition to monogenic causes, PD is also linked to genetic risk variants that predispose to PD but are not causative in and of themselves and may collectively explain 16–36% of the heritable risk of non-monogenic Parkinson’s disease (Göbel et al., 2012; Klein and Westenberger, 2012; Bloem et al., 2021). Several genes appear to be particularly important to our understanding of PD, to the development of model systems that recapitulate cardinal features of PD and to identification of potential novel therapies.

### SNCA (α-Synuclein)

The first gene to be linked to PD was the *SNCA* gene which encodes the protein α-synuclein, a small protein of 16 kilodaltons (KD) (Polymeropoulos et al., 1997). Both mutations in the coding sequence and entire duplications and triplications of the gene that alter gene dosage have been shown to cause PD (Polymeropoulos et al., 1997; Meade et al., 2019; Guo et al., 2021). Thus, *SNCA* is a dosage-dependent gene. The α-synuclein protein that is encoded by *SNCA* is capable of forming higher-order protein multimers and has been shown to be a major component of Lewy bodies and Lewy neurites. Those who inherit duplications or triplications of the *SNCA* gene on chromosome 4 typically present with early-onset PD by the age of 30–40 as shown in a large Swedish pedigree (Fuchs et al., 2007).

**FIGURE 1:**
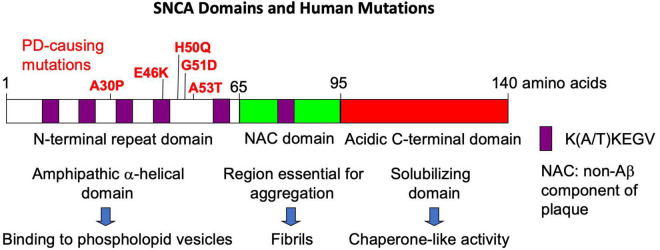
The domains of the SNCA protein and the major human mutations.

The SNCA domains and human mutations are shown in Figure 1. SNCA is an intrinsically disordered protein; however, in the presence of lipid membranes, the N-terminus will take on an alpha helical form (Sung and Eliezer, 2018). The known Parkinson’s disease-causing mutations in SNCA cluster in this N-terminal domain. The middle SNCA domain, historically known as the non-Ab component of plaque (NAC), promotes aggregation (Waxman et al., 2009). Twelve amino acids of this domain are deleted in SNCB, a member of the synuclein protein family that does not aggregate under physiological conditions. SNCA’s acidic C-terminal domain is important for its chaperone-like properties, and its interaction with SNARE complexes involved in vesicular docking (Burré et al., 2010).

The manner by which *SNCA* mutations and copy number variants cause PD is not precisely known (Guo et al., 2021). However, mutations appear to impact the physiological role of the SNCA protein in terms of multimerization and by altering synaptic regulation and maturation, resulting in a reduction in dopamine (DA) in the striatum. In addition, studies also suggest that the SNCA protein may also be involved in other processes such as vesicle endocytosis and degradation, mitochondrial function and/or lysosomal membrane interaction (Benskey et al., 2016).

Although the function of the *SNCA* gene and its encoded protein are still unclear, α-SNCA protein aggregates are commonly observed, post-mortem in PD brain tissue, in structures called Lewy bodies or Lewy neurites (Spillantini et al., 1997). α-SNCA protein is the primary component of Lewy bodies/neurites and has been considered to be a hallmark of PD (Spillantini et al., 1997). Historically, the consensus thought has leaned toward considering the aggregates or inclusions to be a pathogenic species with extensive evidence of transmissibility of protein pathology via injection of SNCA seeds and generation of Parkinson-like neurodegeneration demonstrated (Luk et al., 2012). However, in recent years, a divergent set of data have also suggested that although the aggregates and inclusions are capable of transmitting disease, they may also provide a protective function and the excess of the monomeric form of SNCA protein may also be the pathogenic species or contribute to pathogenesis (Killinger et al., 2019). Similar debates regarding the observation of b-amyloid and tau plagues in brain tissue from those with Alzheimer’s disease (AD) and other neurodegenerative disorders are also occurring, as well (Killinger et al., 2019).

### LRRK2 (Leucine-Rich Repeat Kinase 2)

Other genes with mutations and/or copy number variants that are implicated in PD with substantial evidence include the *LRRK2* gene which encodes a large multidomain protein that contains a *roc* (ras of complex protein) domain and a kinase domain (Li et al., 2014). Monogenic mutations in the gene cause autosomal dominant PD and are associated with variable penetrance from approximately 17% at 50 years of age to 85% by age 70 (Klein and Westenberger, 2012). *LRRK2* gene mutations implicated in PD commonly increase kinase activity (Dächsel and Farrer, 2010); consequently, molecules that are neuroprotective from aberrant LRRK2 activity are sought for both familial *LRRK2-*mutant forms of PD and for other forms that may be characterized by elevated kinase activity (Bloem et al., 2021). Recent data suggests that the target of elevated LRRK2 activity in neurons may be the μ2 subunit of the adaptor protein AP2 (AP2M1), a core component of the clathrin-mediated endocytic machinery (Liu et al., 2021). Phosphorylation cycles by LRRK2 were linked to alterations in endocytosis with subsequent impacts on neuronal survival and demise that recapitulate cardinal features of PD (Liu et al., 2021). Thus, the ability to target a kinase and potentially impact PD provides a rational and novel route in potential therapeutic strategies. Nonetheless, there is a need to consider which patients may benefit from interventions based on *LRRK2* activity and what challenges may be encountered in targeting *LRRK2.*

### DJ-1 (Daisuke-Junko-1)

The *DJ-1* gene is encoded by the *PARK7* locus and was first linked to PD in 2003 (Cookson, 2003; Dawson and Dawson, 2003). DJ-1 protein is a small ubiquitously expressed protein that forms dimers; mutations linked to the *PARK7* locus include insertions, deletions and amino acid substitutions or variants that commonly, but not always, disrupt dimer formation (Dawson and Dawson, 2003). The consequences of mutations in this gene are early onset PD especially the tremor and bradykinesia with other symptoms appearing later and potentially being of a greater severity (anxiety, cognitive decline and psychotic symptoms) (Dawson and Dawson, 2003). The function of the protein remains obscure with some evidence for involvement in α-synucleinopathy, energy utilization, oxidative stress and tau pathology (Dawson and Dawson, 2003; Mani et al., 2021).

### PINK1 (PTEN-Induced Putative Kinase 1)

*PINK1* encodes a kinase of 581 amino acids that also contains a mitochondrial targeting sequence (MTS), transmembrane region (TM), N-terminal regulatory region (NT), N-lobe of the kinase domain, C-lobe of the kinase domain and the C-terminal domain (CTD) (Valente et al., 2004; Liu et al., 2017). The protein is ubiquitously expressed, short-lived and a part of a PINK1:PARKIN protein complex that is required for removal of damaged mitochondria (Valente et al., 2004; Liu et al., 2017). It was first linked to PD in 2004 in a genome wide study of familial PD (Valente et al., 2004) and is thought to provide a protective function to mitochondria during cellular stress by causing the Parkin protein to bind to depolarized mitochondria and induce autophagy (Valente et al., 2004). Mutations in *PINK1* have been linked to accumulation of α-SNCA in Lewy bodies/neurites; in contrast, interaction of PINK1 with α-SNCA may reduce neurotoxicity associated with Lewy bodies/neurites by inducing autophagy in damaged neurons (Valente et al., 2004; Liu et al., 2017). Thus PINK1 and PARKIN may interact to modulate neuronal survival and demise in response to neurotoxicity.

### GBA (GlycosylcerAmidase Beta)

The *GBA* gene encodes the encodes the enzyme glucocerebrosidase (GCase), a lysosomal enzyme that catalyzes the cleavage of a major glycolipid glucosylceramide into glucose and ceramide (Behl et al., 2021). The gene was first implicated in causing Gaucher’s disease (GD) (Sidransky, 2006). However, when GD patients and their families reported for GD care, it was observed that the incidence of PD was much greater than expected with an incidence rate of approximately 25%, a rate that is approximately 20-fold higher than expected (Sidransky, 2006). The pathogenesis associated with *GBA* mutants and PD is thought to be linked to several mechanisms including the failure of GCase to be translocated from the endoplasmic reticulum (ER) and failure to dock with its trafficking transporter protein (Behl et al., 2021).

## Transcription Factors and Induced Neuron Replacement Strategies

Whether genetic factors have been identified or note, the main pathological hallmark of PD is the selective degeneration of midbrain dopamine (mDA) neurons in the substantia nigra (Dauer and Przedborski, 2003; Poewe et al., 2017). Many studies have consequently focused on how these neurons are formed and how to increase their lifespan. Studies using mouse genetic and *in vitro* models have demonstrated that the development and maintenance of mDA neurons are delicately regulated by genetic and signaling networks, including several transcription factors (TFs). During the development of mouse mDA neurons, unique sets of TFs are activated, including Engrailed-1/Engrailed-2, Foxa1/2, Lmx1a/b, Nurr1, Otx2, and Pitx3 (Davidson et al., 1988; Law et al., 1992; Smidt et al., 1997, 2000; Kittappa et al., 2007; Alavian et al., 2008, 2014; Chung et al., 2009; Rodriguez-Traver et al., 2016; Tian et al., 2022). Recent single-cell RNA-seq analysis on developing human brains has confirmed their induction in human mDA neurons as well (Eze et al., 2021). Functional interactions between pairs of these TFs have been identified to ensure proper development of mDA neurons. Specification of mDA neurons progenitors depends on the interaction of signal molecules [e.g., Sonic Hedgehog (SHH) and wingless-type MMTV integration site family, member 1 (WNT1)], growth factors [e.g., glial cell line-derived neurotrophic factor (GDNF), brain-derived neurotrophic factor (BDNF), and fibroblast growth factor 8 (FGF8)], and a series of key TFs including En1/2, Otx2, Lmx1a/b, and Foxa1/2 (Arenas, 2008; Simeone et al., 2011; Joksimovic and Awatramani, 2014; Maury et al., 2015; Brodski et al., 2019). The subsequent differentiation and maturation of mDA neurons requires the expression of Nurr1 and Pitx3, which control the expression of several marker genes for mature mDA neurons, such as TH (tyrosine hydroxylase), DAT (Dopamine active transporter, encoded by the SLC6A3 gene) and VMAT2 (vesicular monoamine transporter 2), AADC (DOPA decarboxylase), DRD2 (dopamine receptor D2), or ALDH1A1 (aldehyde dehydrogenase 1 family, member A1) (Tian et al., 2022).

These TFs remain present in adult mDA neurons and are required for long-term survival of mDA neuron (Alavian et al., 2008, 2014; Tian et al., 2022). Importantly, disruption of one of these TFs in mouse genetic models displays several features reminiscent of PD. Loss of En1/2, although mDA neurons are initially generation, leads to progressive demise of mDA neurons in the ventral midbrain starting from 6 weeks of age. These mice also develop Parkinson disease-like symptoms that is related to mitochondrial dysfunction and retrograde degeneration (Alberi et al., 2004; Sonnier et al., 2007; Alvarez-Fischer et al., 2011; Haubenberger et al., 2011; Fuchs et al., 2012). Similar phenotype has been reported in Lmx1b conditional knockout mice (Smidt et al., 2000; Bergman et al., 2009). In addition, conditional adult ablation of Nurr1 or Foxa1/2 leads to a decline of striatal DA levels in substantia nigra of aged animals (Zetterström et al., 1997; Domanskyi et al., 2014).

Interestingly, some of the TFs, especially homeoproteins, can transduce cells and provide neuroprotection in PD models (Blaudin de The et al., 2016). Recombinant En1/2 proteins can rescue mDA neuronal loss and increase physiological activities of mDA neurons in several PD models, including mutated En1 or alpha-synuclein mice, and chemically induced PD models [e.g., MPTP, rotenone, 6-OHDA (6-hydroxydopamine)] (Pen et al., 2008; Alvarez-Fischer et al., 2011). Moreover, overexpression of Otx2 can also revent the progressive loss of mDA neurons in substantia nigra in En1 ± mice and MPTP PD mice models (Ibad et al., 2011; Giovannantonio et al., 2013). These results highlight the potential of TF transduction as a potential strategy for PD treatment.

### Direct Conversion of Fibroblasts to Induced Neurons

Studies outlined above underline the role of TFs in development and also suggest the potential for use in cell replacement therapy for PD. Indeed, mDA-related TFs not only coordinate the development and survival of mDA neurons *in vivo* but are also able to guide *in vitro* cell fate conversion of various cell types into mDA neurons (Tian et al., 2022). Mouse and human fibroblasts can be efficiently directed to generate induced DA (iDA) neurons by the combined action of the Ascl1, Nurr1, and Lmx1a TFs (Caiazzo et al., 2011). Forced expression of Lmx1a or various combinations of TFs is also able to induce differentiation of human pluripotent stem cells into mDA neurons *in vitro* (Andersson et al., 2006; Friling et al., 2009; Panman et al., 2011; Flitsch et al., 2020; Tian et al., 2022). In addition, several findings indicate that NURR1 alone or in combination with other TFs, such as FOXA2, NGN2, ASCL2, and PITX3, induces iDA neurons from hPSCs or human astrocytes or fibroblasts (Rodriguez-Traver et al., 2016). More recently, several groups have reported direct cell fate conversion *in vivo* triggered by TFs with the overexpression of Ascl1, Lmx1a, Neurod1, and miRNA-218 in mouse brain directly converting astrocytes to mDA neurons that when transplanted, result in marked improvement in spontaneous motor behavior deficits in 6-OHDA-induced PD model mice (Rivetti di Val Cervo et al., 2017).

Clearly, the elucidation of the roles and mechanisms of action of TFs during development and adulthood could bring important insights into PD pathogenesis and suggest new therapeutic strategies. Nonetheless, there is a need for animal models systems that recapitulate the overall pathology, genetic risk and therapeutic potential of PD in men and women.

## Cellular Models of Parkinson’s Disease

Cellular models of PD fall into two broad categories: Non-patient specific human cell lines and patient-specific human cell lines (Schüle et al., 2009). The non-patient specific cellular models were developed earlier, in the 1990’s to early 2000’s, and include human neuroblastoma cell lines, embryonic carcinoma cell lines, immortalized human embryonic mesencephalic cells, differentiated human embryonic stem cell lines and human mesenchymal stem cell lines (Schüle et al., 2009). These cell lines are useful in many types of studies as they have characteristic expression of tyrosine hydroxylase, dopamine receptors (2 and 3) upon differentiation, express many late markers of mature dopaminergic neurons, and have enhanced susceptibility to oxidative stressors, may in some cases upregulate expression from *SNCA* and synaptic protein genes, and recapitulate electrophysiological characteristics of mature DA neurons (Schüle et al., 2009). Patient-specific cell lines include the first generation cybrid cell lines, primary human fibroblast cell lines and induced pluripotent stem cell (iPSC) lines (Schüle et al., 2009; Byers et al., 2011; Nguyen et al., 2011; Zhang et al., 2014; Stoddard-Bennett and Pera, 2019). Subsequently, the field has moved forward significantly with special collections of integration-free lines such as those derived from Parkinson’s disease patients carrying mutations in the GBA1 gene have also been produced (Rodriguez-Traver et al., 2019). Recently, a compendious summary of iPSC lines established in the first decade of iPSC biomedical research was published (Ren et al., 2019). Notably, lines have been developed that carry mutations in *LRRK2, SNCA, GBA, PINK1*, and *Parkin.* The phenotypes that appear to be faithfully recapitulated in the lines include morphological abnormalities, defects in autophagy/mitophagy, altered a-SNCA protein expression and/or accumulation, mitochondrial dysfunction, nuclear abnormalities, altered synaptic transport, oxidative stress and susceptibility to cellular stressors (Ren et al., 2019). Moreover, several studies have now reported success in pharmaceutical testing or validation studies using iPSC models (Ren et al., 2019).

## Animal Models of Parkinson’s Disease

As noted in a recent review and evident upon inspection of the biomedical research enterprise, there are three commonly used animal groups for modeling human disease: Rodents, non-human primates and non-mammalian species (Konnova and Swanberg, 2018). Each of these groups has distinct advantages and disadvantages as outlined below and as reviewed by Konnova and Swanberg (2018).

Rodents, especially mice and rats, have been studied across biomedical fields and are convenient to care for, relatively inexpensive compared to non-human primates, well-characterized genetically and easily genetically modified. The majority of >23,000 studies in animals over the last three decades or more have involved rodents (48% of studies include rats and 37% include mice); in contrast, only approximately 10% of studies included non-human primates and 5% include non-mammalian species (Konnova and Swanberg, 2018). These studies often model the genetics of PD by humanization of key loci and subsequent chemical or surgical lesion of distinct brain regions as in a recent study of the impact of α-synuclein pathology on transplanted hESC-derived dopaminergic neurons in a humanized α-synuclein rat model of PD (Hoban et al., 2020).

Non-human primates (NHPs), of course, may provide additional insights into PD pathology given the anatomic and genetic similarities to humans. This clear advantage is associated, however, with the obvious increase in costs of care and housing, complexity of ethical concerns, and longer lifespan (Konnova and Swanberg, 2018). Fewer than 10% of animal studies of PD include NHPs and those that do are typically a necessary step toward clinical validation of a potential therapeutic strategy or pharmaceutical agent. The most commonly used NHP is the macaque. PD symptoms are often induced with neurotoxins and/or viral vector-mediated gene expression (Konnova and Swanberg, 2018).

Finally, approximately 5% of animal studies of PD use non-mammalian species especially the fruit fly, *Drosophila melanogaster*, and the round worm, *Caenorhabditis elegans* (Konnova and Swanberg, 2018). Both *Drosophila* and *C. elegans* have the advantages of low cost to maintain, large number of progeny, short life span and ability to conduct large-scale screens (Konnova and Swanberg, 2018). Less-widely used are various species of fish especially the zebrafish, *Danio rerio*, which has been used in a wide variety of high throughput molecular screens and appears to recapitulate many of the late-onset PD symptoms remarkably well (Kodera and Matsui, 2022). The primary disadvantage of non-mammalian species is the obvious differences in physiology and biology between these species and humans.

## Summary

Parkinson’s disease is the second most common human neurodegenerative disease with a prevalence that is increases as the lifespan of men and women around the globe has increased over the last several decade. The genetics, underlying molecular biology, cell-based systems and animal models to probe etiology and therapeutic strategies have improved markedly over the last two decades. Yet, the details regarding the development of dopaminergic neurons and their ultimate demise have not yet been assembled. In the upcoming decade, it will be critical to expand our studies of personalized medicine or interventions that may more appropriately reflect genetic status, physiology and key characteristics of individual PD patients. Moreover, it will be beneficial to consider the non-neuronal phenotypes associated with PD, as well, in order to address those consequences.

## Author Contributions

All authors listed have made a substantial, direct, and intellectual contribution to the work, and approved it for publication.

## Conflict of Interest

DC and RR were employed byMcLaughlin Research Institute for Biomedical Sciences, Inc. The remaining authors declare that the research was conducted in the absence of any commercial or financial relationships that could be construed as a potential conflict of interest.

## Publisher’s Note

All claims expressed in this article are solely those of the authors and do not necessarily represent those of their affiliated organizations, or those of the publisher, the editors and the reviewers. Any product that may be evaluated in this article, or claim that may be made by its manufacturer, is not guaranteed or endorsed by the publisher.
